# Author Name Disambiguation for PubMed

**DOI:** 10.1002/asi.23063

**Published:** 2013-11-21

**Authors:** Wanli Liu, Rezarta Islamaj Doğan, Sun Kim, Donald C. Comeau, Won Kim, Lana Yeganova, Zhiyong Lu, W. John Wilbur

**Affiliations:** National Center for Biotechnology Information, National Library of Medicine, National Institutes of Health, 8600 Rockville Pike, Bethesda MD, 20894

## Abstract

Log analysis shows that PubMed users frequently use author names in queries for retrieving scientific literature. However, author name ambiguity may lead to irrelevant retrieval results. To improve the PubMed user experience with author name queries, we designed an author name disambiguation system consisting of similarity estimation and agglomerative clustering. A machine-learning method was employed to score the features for disambiguating a pair of papers with ambiguous names. These features enable the computation of pairwise similarity scores to estimate the probability of a pair of papers belonging to the same author, which drives an agglomerative clustering algorithm regulated by 2 factors: name compatibility and probability level. With transitivity violation correction, high precision author clustering is achieved by focusing on minimizing false-positive pairing. Disambiguation performance is evaluated with manual verification of random samples of pairs from clustering results. When compared with a state-of-the-art system, our evaluation shows that among all the pairs the lumping error rate drops from 10.1% to 2.2% for our system, while the splitting error rises from 1.8% to 7.7%. This results in an overall error rate of 9.9%, compared with 11.9% for the state-of-the-art method. Other evaluations based on gold standard data also show the increase in accuracy of our clustering. We attribute the performance improvement to the machine-learning method driven by a large-scale training set and the clustering algorithm regulated by a name compatibility scheme preferring precision. With integration of the author name disambiguation system into the PubMed search engine, the overall click-through-rate of PubMed users on author name query results improved from 34.9% to 36.9%.

## Introduction

With the fast growth of scientific literature, author name ambiguity has become a critical issue in managing information at the individual level. Due to the lack of universal standards for name information, there are two aspects of name ambiguity: name synonymy (a single author with multiple name representations), and name homonymy (multiple authors sharing the same name representation). In practice, name synonymy and homonymy cause different problems in information retrieval. For authors with synonymous names, it is hard to retrieve relevant information at high recall without complete knowledge of various name representations shared by the same author. On the other hand, queries with a homonymous name often lead to irrelevant information retrieval from other authors sharing the same name. Therefore, ambiguous names pose a serious challenge to the efficacy of a modern bibliographic retrieval system aimed at both high precision and high recall.

There have been many efforts to study author name ambiguity and solutions have been proposed in numerous fields (Smalheiser & Torvik, 2009). Libraries have developed and maintained authorship control with manual curation, such as the Library of Congress. Recently, some web services have encouraged users to help with the disambiguation task at the community level (DBLife) or the individual level (Community of Science) by inviting authors to register their publications online (orcid.org, authorclaim.org, Google Scholar) or assigning a unique ID (Thomson Reuters ResearcherID). Compared to the high cost of centralized manual authorship control, community level efforts involving authors are more efficient. However, voluntary participation can only benefit from active authors, with accuracy and coverage subject to the motivation and integrity of the participants. Recently, some automatic methods have been proposed to provide less costly and more efficient solutions (Huang, Ertekin, & Giles, 2006; Song, Huang, Councill, Li, & Giles, 2007; Torvik & Smalheiser, 2009; Treeratpituk & Giles, 2009). There are also various applications strongly related to author name disambiguation, such as word sense disambiguation, topic modeling, research assessment, collaboration networks, reference analysis, and stylometry, as recently reviewed (Smalheiser & Torvik, 2009; Elliot, 2010; Ferreira, Gonçalves, & Laender, 2012). These applications can benefit the name resolution task by providing important information other than conventional features extracted from metadata (Lin & Wilbur, 2007; Yin, Han, & Yu, 2007; Varol, Stafford, Kovvuri, & Chitti, 2010; Bernardi & Le, 2011). On the other hand, high-quality name disambiguation can also benefit these applications. The demand from these related applications strongly implies the importance of effective author name disambiguation.

**FIG. 1:**
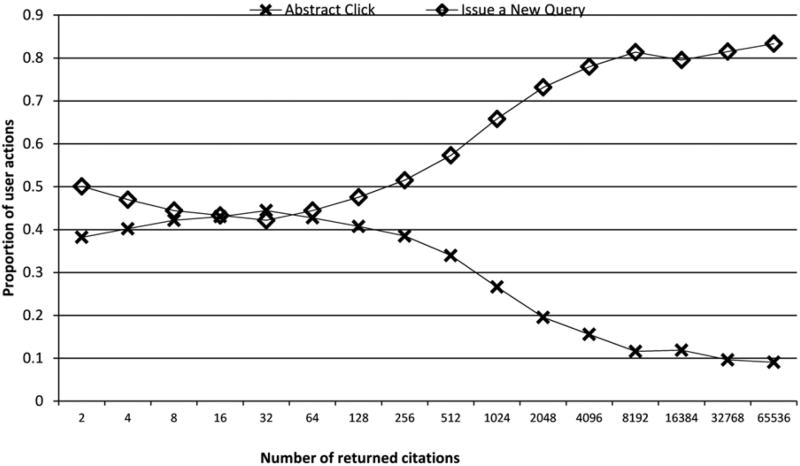
User behavior statistics with different number of retrieved citations.

As the entry point to the large-scale National Library of Medicine database for biomedical literature, the PubMed engine provides online access to more than 22 million scientific citations. Millions of users search this biomedical database through PubMed every day. An analysis of the PubMed web log in 2009 showed that about 36% of the queries contain an author name (Islamaj Dogan, Murray, Névéol, & Lu, 2009). Out of more than 79 million names in the PubMed collection, more than 10% will lead to retrieval of over 100 citations that are authored by multiple authors. PubMed user behavior statistics indicate that users tend to issue a new query with additional details rather than click through to an abstract as a response to a large number of citations retrieved for an author name query, as shown in Figure 1.

To improve the users’ search experience and facilitate other related applications, we have implemented author name disambiguation for PubMed citations. Our system provides an up-to-date author name disambiguation function for a large-scale biomedical literature information retrieval system. Motivated by previous approaches, our implementation is based on a machine-learning method, which is trained on features extracted from various fields of PubMed citations. Our system makes two unique contributions to the field. First, instead of using the author name to be disambiguated as a source of features for machine learning, we propose a name compatibility scheme to regulate the relationship between citations sharing similar name variants in creating training data and producing final clustering results. Second, to learn the feature weighting for PubMed metadata, we design an unsupervised method to automatically create large-scale training data sets. Positive training data are based on the low-frequency author names, which with high probability represent the same individual. Negative data are randomly generated. The positive training data are filtered with name compatibility and a publication year check to further improve the label accuracy. Considering namespace size and the expected proportion of positive pairs, similarity scores are converted to pairwise probabilities with the pool adjacent violators (PAV) algorithm (Zadrozny & Elkan, 2002; Wilbur, Yeganova, & Kim, 2005). With a clustering priority ordering scheme combining both name compatibility and probability level, the clustering algorithm produces final clustering results by focusing on the highest quality initial clustering and minimizing false-positive clustering.

The details of our presentation are laid out as follows: Related Work focuses on discussing comparable techniques proposed in the past and justifying our technical decisions; the Materials and Methods section presents a detailed description of training set generation, machine-learning methods, and the clustering algorithm; the Results and Discussion sections evaluate the advantage of our technique through a number of experiments and comparisons; and the Conclusion section presents conclusions and outlines future work.

### Related Work

Due to the limitations of manual authorship management, numerous recent name disambiguation studies focus on automatic techniques for large-scale literature systems. To process large-scale data efficiently, it is necessary to define the scope (*block*) of author name disambiguation appropriately to minimize the computation cost without losing significant clustering opportunities. A block is a set of name variants that are considered candidates for possible identity. Several blocking methods handling name variants are discussed (On, Lee, Kang, & Mitra, 2005) to find the appropriate block size. Collective entity resolution (Bhattacharya & Getoor, 2007) shows that clustering quality can be improved at the cost of additional computational complexity by examining clustering beyond block division. In our work, a block (*namespace*) consists of citations sharing a common last name and first name initial.

Normally disambiguation methods estimate authorship relationships within the same block by clustering similar citations with high intercitation similarity while separating citations with low similarity. The intercitation similarities are estimated based on different features. Some systems use coauthor information alone (On et al., 2005; Kang, Na, Lee, Jung, Kim, et al., 2009). Some systems combine various citation features (Han, Zha, & Giles, 2005; Torvik, Weeber, Swanson, & Smalheiser, 2005; Soler, 2007; Yin et al., 2007), and some combine citation features with disambiguating heuristics based on predefined patterns (Cota, Ferreira, Nascimento, Gonçalves, & Laender, 2010). Besides conventional citation information, some works (Song et al., 2007; Yang, Peng, Jiang, Lee, & Ho, 2008; Bernardi & Le, 2011) also exploit topic models to obtain features. McRae-Spencer and Shadbolt (2006) include self-citation information as features and Levin et al. (2012) expand features to the citation information between articles. Similar to some previous works, the features in our machine-learning method are also created from PubMed metadata which are strongly indicative of author identity. However, instead of using the name to be disambiguated as part of the similarity profile, we use author name compatibility in restricting the clustering process.

Numerous methods have been developed to convert authorship features to intercitation distance. In unsupervised learning (Mann & Yarowsky, 2003; Ferreira, Veloso, Gonçalves, & Laender, 2010), extraction patterns based on biographic data and recurring coauthorship patterns are employed to create positive training data. Supervised machine-learning approaches require training data sets to learn the feature weighting (Han, Giles, Zha, Li, & Tsioutsiouliklis, 2004; On et al., 2005; Torvik et al., 2005; Treeratpituk & Giles, 2009). Some supervised methods (Han et al., 2004; On et al., 2005; Huang et al., 2006) train machine-learning kernels such as SVM with training data to find the optimal separating hyperplane and perform feature selection as well. In Treeratpituk and Giles (2009), a Random Forest approach is shown to benefit from variable importance in feature selection, which helps to outperform other techniques. We use a supervised machine-learning method to characterize the same-author relationship with large training data sets. Our positive training data are assembled based on the assumption that a rare author name generally represents the same author throughout, similar to the construction of matching data in Torvik and Smalheiser (2009). However, our training data sets are much larger and the positive data are filtered with a name compatibility check and a publication year check to minimize false-positive pairs.

Typical methods for computing authorship probability include computing similarity based on term frequency (On et al., 2005; Treeratpituk & Giles, 2009), and estimating the prior probability from random samples (Soler, 2007; Torvik & Smalheiser, 2009). In our work, the similarity profile for a citation pair is converted to a pairwise probability with the PAV algorithm. In this process, both name frequency and a prior estimation of the proportion of positive pairs play an important role.

Name disambiguation is implemented by assigning citations to a named author based on the relationship among citations, usually during a clustering process. Various clustering algorithms include agglomerative clustering with intercitation distance (Mann & Yarowsky, 2003; Culotta, Kanani, Hall, Wick, & McCallum, 2007; Song et al., 2007; Kang et al., 2009; Torvik & Smalheiser, 2009), K-way spectral clustering (Han et al., 2005), boosted-tree (Wang, Berzins, Hicks, Melkers, Xiao, et al., 2012), affinity propagation (Fan, Wang, Pu, Zhou, & Lv, 2011), quasi-cliques (On, Elmacioglu, Lee, Kang, & Pei, 2006), latent topic model (Shu, Long, & Meng, 2009), and density-based spatial clustering of applications with noise (DBSCAN) (Huang et al., 2006). To minimize the impact of data noise on clustering quality, transitivity violations of pairwise relationships are corrected in DBSCAN (Huang et al., 2006) and with a triplet correction scheme in (Torvik & Smalheiser, 2009). To correct transitivity violations, we apply a correction scheme similar to Torvik and Smalheiser (2009) with a stronger boosting for false-negative pairs. As previously (Mann & Yarowsky, 2003; Torvik & Smalheiser, 2009), an unsupervised agglomerative clustering algorithm disambiguates citations within a namespace. As pointed out before (Mann & Yarowsky, 2003; Tang, 2012), clustering the most similar citations first can help create a high-quality clustering result. In addition to ordering clustering by similarity level, our clustering process is also regulated by another ordering scheme based on name compatibility, which schedules merging clusters with closer name information at earlier stages.

## Materials and Methods

**FIG. 2:**
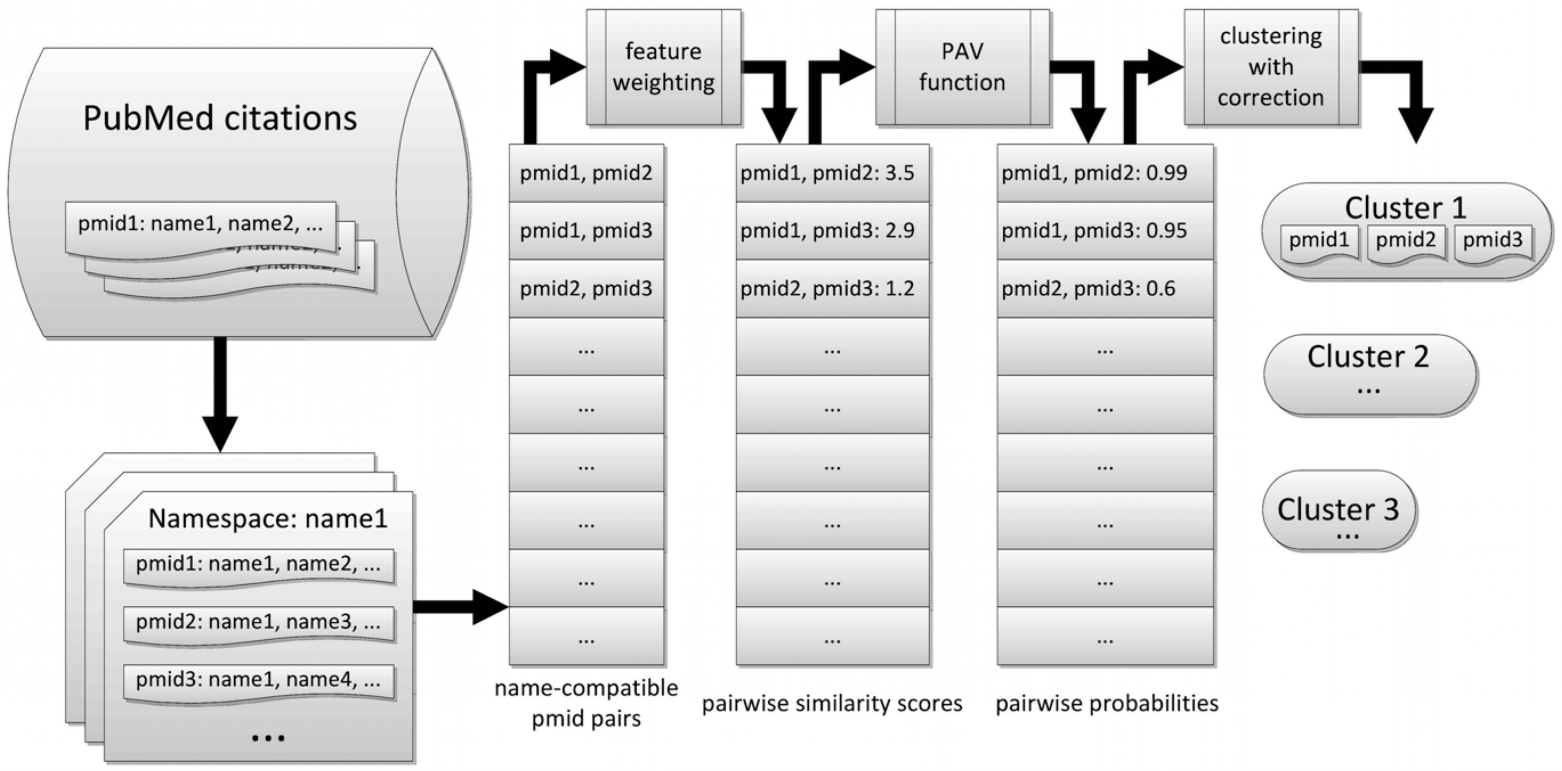
Workflow of similarity computation and clustering.

A popular framework adopted by successful author name disambiguation techniques (Torvik & Smalheiser, 2009; Wang et al., 2012) is clustering citations based on pairwise intercitation probabilities. First, all the citations sharing common author names to be disambiguated are grouped into namespaces. Then all the citations satisfying our name compatibility check within each namespace are compared with one another to measure overall intercitation similarity scores based on machine-learned feature weighting. The intercitation similarity scores are then transformed into pairwise probabilities through a PAV function, and then delivered to the clustering algorithm where correction of transitivity violations is performed. Similar citations are clustered into different partitions with maximum likelihood, with each partition of citations expected to belong to an individual author. This process is illustrated in Figure 2 and explained in the following sections.

### Processing Names in PubMed

PubMed provides online access to the National Library of Medicine database (MEDLINE) and other publications, which contain more than 22 million scientific citations from 1865 to the present. A small fraction (2.4%) of all PubMed citations has an empty author field. The rest of the PubMed citations, which are the focus of this work, list authors in the same order as the publication. There were more than 76 million name instances in ~22 million citations in 2012. Except for a small number of cases with only a last name, the names in the author field are formatted as last name, first name, or first name initial, with middle names or middle name initials also available for a minority of cases.

Some issues have to be addressed before author names can be processed appropriately. The first issue is how to properly handle names encoded in Unicode other than ASCII characters. As a global database, PubMed collects publications by authors from all around the world in a variety of languages. Many authors choose to use different character encodings for the representation of their names in various situations. For example, German authors may use German characters for their names in German journals while converting the German characters to English characters for an English publication. Normally, PubMed collects publications of different languages in Unicode encoding. In this work, to unify the same name in different characters, all non-ASCII characters in author names are converted to their corresponding ASCII characters using a mapping table we developed based on the database from the Unicode Consortium (http://www.unicode.org). A number of Unicode characters are represented as named and numeric HTML entities, and they are also converted to ASCII characters, or removed if no ASCII character can be found. With all names unified with an ASCII character representation, the names in disambiguation results are mapped back to their original encoding for the purpose of PubMed indexing.

The second issue has to do with the structure of the author field. Normally the authors are organized as a single list, and each author appears only once. However, some publications involving a large number of collaborating authors tend to group authors according to their contribution to the work. For example, the Alliance for Cellular Signaling is a large-scale cross-discipline and cross-institution collaboration involving dozens of investigators and scientists serving on different committees. An article (PMID: 12478301) published in *Nature* lists members of each committee, while Adam P. Arkin serves on both the Steering Committee and Editorial Committee. In the PubMed database, this structured author list is flattened. As a result, Adam P. Arkin appears twice in the author field. The assumption that an individual author appears only once in the author field would end up treating those two instances of Adam P. Arkin wrongly as two individuals. However, currently this issue has not been addressed. It would require either referencing the original publication, or manually detecting and correcting these errors individually in the database.

The third issue in the name field concerns nonauthor entities. For example, some organizations publish reports under their institutional names (e.g., American Pharmacists Association). Although they are not individual author names, they are still treated as individual authors, with the institutional name as last name without first or middle name information.

Based on the author name instances found in the PubMed database, all the citations are organized into *namespaces* in a similar blocking fashion as (On et al., 2005). The *namespace* is defined as follows:

Given a last name (*LN*) and a first name initial (*FNI*), a namespace consists of all the PubMed citations, in whose author field one or more author names are compatible with (*LN*+*FNI*).

Any two names *NAME_i_* and *NAME_j_* are mutually *compatible* if the following conditions hold for at least one ordering of (*i, j*):

1. *NAME_i_.last_name* == *NAME_j_.last_name*
2. *NAME_i_.first_name* == *NAME_j_.first_name*
  or *NAME_i_.first_name.first_character* == *NAME_j_.first-_name* (the first name information for NAME_j_ is only initial letter)
  or *NAME_i_.first_name* == NULL
  or *NAME_i_*.first_name == *NAME_j_*.first_name.variant
3. *NAME_i_.middle_name* == *NAME_j_.middle_name*
  or *NAME_i_.middle_name.first_character* == *NAME_j_.middle_name*
  or *NAME_i_.middle_name* == NULL

The name compatibility check allows two names potentially belonging to the same author to be considered in the disambiguation process. Any two names failing the compatibility check are regarded as belonging to different authors. Note that some authors do change their names due to various reasons (e.g., marriage), and it requires external information to relate these incompatible names, which is not implemented in this work. As shown in Torvik and Smalheiser (2009), name variants such as nicknames and partially matching names should be considered in the name compatibility check. We built a name variant map based on the database from the USGenWeb Project (http://www.usgenweb.org) to support variant matching for first names sharing common initials in the compatibility check. We also supplement this map with other variants found in PubMed.

According to the definition of name compatibility, one namespace includes all the citation members sharing author names compatible with a common last name and first name initial. For example, the citations with names such as J. Miller, Joel S. Miller, and Judith Miller all belong to the namespace MILLER+J, since they all share the same last name and first name initial.

Note that the cases with only a last name (without first or middle name) are grouped into a separate namespace. Although this case is compatible with any name having the common last name and any first or middle name, we place it into a separate namespace to avoid oversize namespaces and unnecessary computation. In total, 3.88 million namespaces are created, and 2.43 million namespaces own more than one citation and require name disambiguation.

### Features for Machine Learning

Designated with a distinct PMID (PubMed ID number), each PubMed citation contains a number of fields for metadata, providing information for title, journal name, publication date, authors, affiliation, abstract, MeSH (Medical Subject Headings) terms, substance, grant, etc. The availability varies for each field, as shown in Table 1.

For historic reasons, availability is low for some fields. Since the metadata fields provide important evidence for name disambiguation tasks, the lack of key metadata could result in a poor disambiguation outcome. With publishers providing more metadata with more frequent updating, recent citations have higher metadata availability. Recently, new information has also been added to complement traditional metadata. For example, some corresponding authors provide personal e-mail addresses, which normally appear as part of the affiliation field. Previous work (Torvik et al., 2005) shows that personal information (i.e., e-mail and author web page) improves the disambiguation accuracy.

Different PubMed fields are not equally significant in gauging similarity. To determine how each field contributes to the overall intercitation similarity, machine-learning methods are applied on positive and negative training data sets. The positive data set contains sample pairs of citations by the same author, while the negative data set contains sample pairs of citations by different authors. Ideally, manual author name disambiguation is required to create positive and negative training samples for PubMed citations. However, it is prohibitively time-consuming to prepare large size training data sets. Instead, we observe that citations from small namespaces tend to belong to the same author. To obtain high-quality training data sets for machine-learning methods, we take advantage of this observation to approximate true authorship relations.

Our positive data set includes about 6.7 million sample pairs from namespaces with two to five citations. Although machine-learning algorithms benefit from a large number of positive samples, their performance can be compromised by potential false-positive samples. To minimize the impact of such imperfect pairs, the pairs with incompatible names (identical last name and first initial but conflicting first names) are excluded, as well as the pairs of documents with publication dates over 50 years apart. For the negative data set, about 6.5 million sample pairs are randomly selected as pairs of citations from different namespaces. Each sample pair is represented by a set of features, from which its similarity is estimated. This computation is based on the contents of the nine fields as summarized in Table 2. The nine fields were selected as features because they are highly indicative of author identity. Among these nine fields, the Date field is represented by the publication year. The feature for this field is computed as the absolute difference in publication year of the pair of citations:

$$\text{year}\_\text{diff}=\text{abs}({\text{year}}_{1}-{\text{year}}_{2}).$$

After a stopword list based on PubMed general stopwords (http://www.nlm.nih.gov/bsd/disted/pubmedtutorial/020_170.html) is applied, each of the remaining eight text fields is individually processed to create a similarity score with the conventional IDF (inverse document frequency) (Spärck Jones, 1973) computation using statistics from different fields. At the corpus level, *N* denotes the total number of all the PubMed citations. Each field *F* is composed of tokens *f*, and we use *n_f_* to denote the number of citations containing the term *f*. Suppose we have a pair of citations *D_1_* and *D_2_*, with their token sets *T_1_* and *T_2_* from *F*. The pairwise similarity computation starts by producing a set of common tokens *C* = *T_1_*∩*T_2_*. The sum of *idf* of the common terms is computed as:

$$\text{idf}\_{\text{sum}}_{C}=\sum_{f\in C}\text{log}\frac{N}{n_{f}}.$$

Similarly, the sum of *idf* over all the unique terms in each citation is computed as:

$$\text{idf}\_{\text{sum}}_{T_{i}}=\sum_{f\in T_{i}}\text{log}\frac{N}{n_{f}}\;(i=1,2).$$

To measure the relative significance of common terms for each citation, the *idf* sum is normalized by the sum of *idf* of all tokens:

$$\text{idf}\_{\text{sum}}_{{\text{norm}}_{i}}=\frac{\text{idf}\_{\text{sum}}_{C}}{\text{idf}\_{\text{sum}}_{T_{i}}}\;(i=1,2).$$

The overall pairwise similarity score is then computed as the average of the normalized *idf* sum of the citations:

$${\text{similarity}}_{\text{overall}}=\frac{\text{idf}\_{\text{sum}}_{\text{norm}1}+\text{idf}\_{\text{sum}}_{\text{norm}2}}{2}.$$

The author field is a special text field, and is handled differently. The two citations are considered to share a common name if a name appears in compatible forms in the two citations. For positive samples, which can have multiple common names, a randomly chosen common name is excluded from the *common coauthors* set and both *all authors* sets. For negative samples, which are chosen from different namespaces, the author names qualifying for each namespace are excluded from each *all authors* set. Like other text fields, the similarity score for the author field is valued in the range from 0 to 1.

$${\text{similarity}}_{\text{name}}\;=(\frac{\sum_{\text{common coauthors}}\text{log}\frac{N}{n_{f}}}{\sum_{\text{all authors}1}\text{log}\frac{N}{n_{f}}}+\frac{\sum_{\text{common coauthors}}\text{log}\frac{N}{n_{f}}}{\sum_{\text{all authors}2}\text{log}\frac{N}{n_{f}}})/2$$

The positive and negative training sets are employed to train a wide margin classifier with a variation of quadratic loss function, the modified Huber loss function (Zhang, 2004; Smith, Kim, & Wilbur, 2012). The Huber classifier analyzes the similarity scores from all the PubMed fields for each sample in both positive and negative training sets, and creates overall Huber score for each sample as the sum of weights of all fields (see Table 2).

$$\text{Huber Score}=\sum_{i=1\sim 9}{\text{weight}}_{i}({\text{similarity}}_{i})$$

**FIG. 3:**
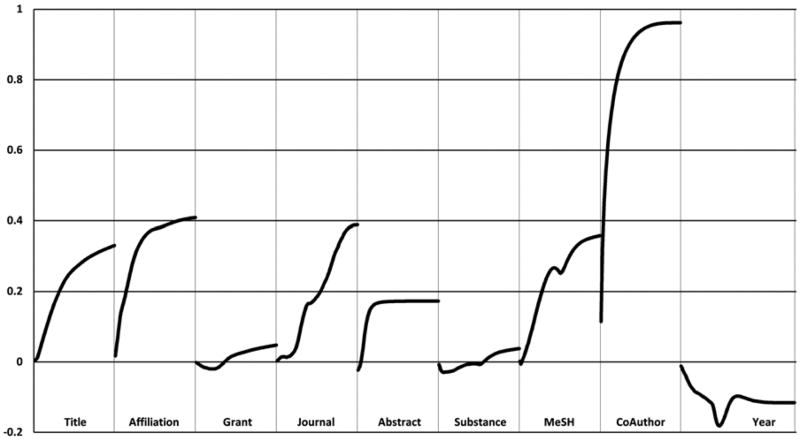
Weight functions of PubMed field features.

Each field is assigned with a weight function which converts its similarity score to a weight. The Huber classifier learns these weight functions, to achieve overall optimal classification for the training data. The weight functions for all the PubMed fields are shown in Figure 3. For each of the first eight text fields, the X axis indicates the similarity score *similarity_i_* in the range from 0 to 1, and the corresponding Huber score weight via weight function *weight_i_*(*similarity_i_*) is shown along the Y axis. For the Date field similarity score, the absolute publication year difference between two citations ranges up to 200.

The weight functions reveal the significance of each field in the machine-learning process. Generally, text fields contribute positively to the Huber score, while the publication Year difference makes negative contributions, which suggests that the greater the disparity in publication dates, the less likely the two papers have the same author. Among the text fields, the Author field has the greatest positive impact on the overall Huber score. Other text fields, such as Title, Affiliation, Journal, and MeSH fields are similarly important in measuring pairwise similarity. Lower-weighted than other text fields, the Abstract field normally consists of significantly more terms, many of which are not strongly associated with author identity, and hence are not as informative. For the Grant and Substance fields, a small amount of similarity is actually evidence of a different grant or a different substance, and hence evidence against having the same author. For example, the grant information from different researchers might only match on the token “NIH.” Consequently, the similarity scores for these two fields have to be relatively high to make a positive contribution to the overall Huber score. The Huber classifier achieves average precision of 97.3% in a 10-fold cross-validation test over the training data. This high precision is strong support for the assumption that low frequency namespaces are almost completely positive data. We also observe that a smaller training set leads to the degradation of precision. In classification with different sizes of training data and the same test data, the precision starts to decrease noticeably when the training data shrink by a factor of 100, and drops by more than 1% when the training data shrink by a factor of 1,000. The weight functions resulting from smaller training sets become rough and irregular (compared to Figure 3), and this signals overtraining.

Recently, some techniques have been proposed to train on a large number of samples, that is, the probabilistic similarity metric (Torvik et al., 2005), bootstrapping (Levin, Krawczyk, Bethard, & Jurafsky, 2012). The probabilistic similarity metric is based on the pairs of citations created by examining if they share matching names. In bootstrapping, a set of high-precision rules are applied to search for positive samples, as well as negative rules to detect negative samples, with some rules based on external information (self-citation). While these techniques (i.e., name matching and name rule) work in a similar fashion to our name compatibility check, the high-precision sample-selecting rules tend to cherry-pick the positive samples with the features reflected in the rules and ignore other information indicating the same-author relationship. Our training set construction is able to collect positive cases without unfairly biasing towards features which strongly indicate the same-author relationship and this allows us to conduct machine learning in a more balanced fashion.

### Converting Huber Score to Probability

Similarity scores computed by the Huber classifier need to be converted to pairwise probabilities of same author to be used in the clustering algorithm. This allows a fair comparison of pairwise similarity among different pairs. Given a Huber score *s*, the PAV algorithm (Zadrozny & Elkan, 2002; Wilbur et al., 2005) can estimate its probability *p* of representing a positive (same author) pair. The PAV algorithm produces a monotonically nondecreasing function *p(s)*, which assigns a higher probability *p(s)* to a higher score *s*. The resulting probability estimation is then employed to represent similarity between the pair of citations in the clustering computation for a namespace. Because prior probabilities need to be considered, *p(s)* needs to be tuned for each namespace.

Creation of PAV functions requires high-quality data containing positive and negative data points faithfully reflecting the distribution of Huber scores. A data set of precise same-author relationships is constructed by collecting self-citation pairs. The positive data set totals 2.7 million such pairs sampled within a time window of over 30 years. For the negative data set, we reuse the same 6.5 million pair negative data set constructed for machine learning.

**FIG. 4:**
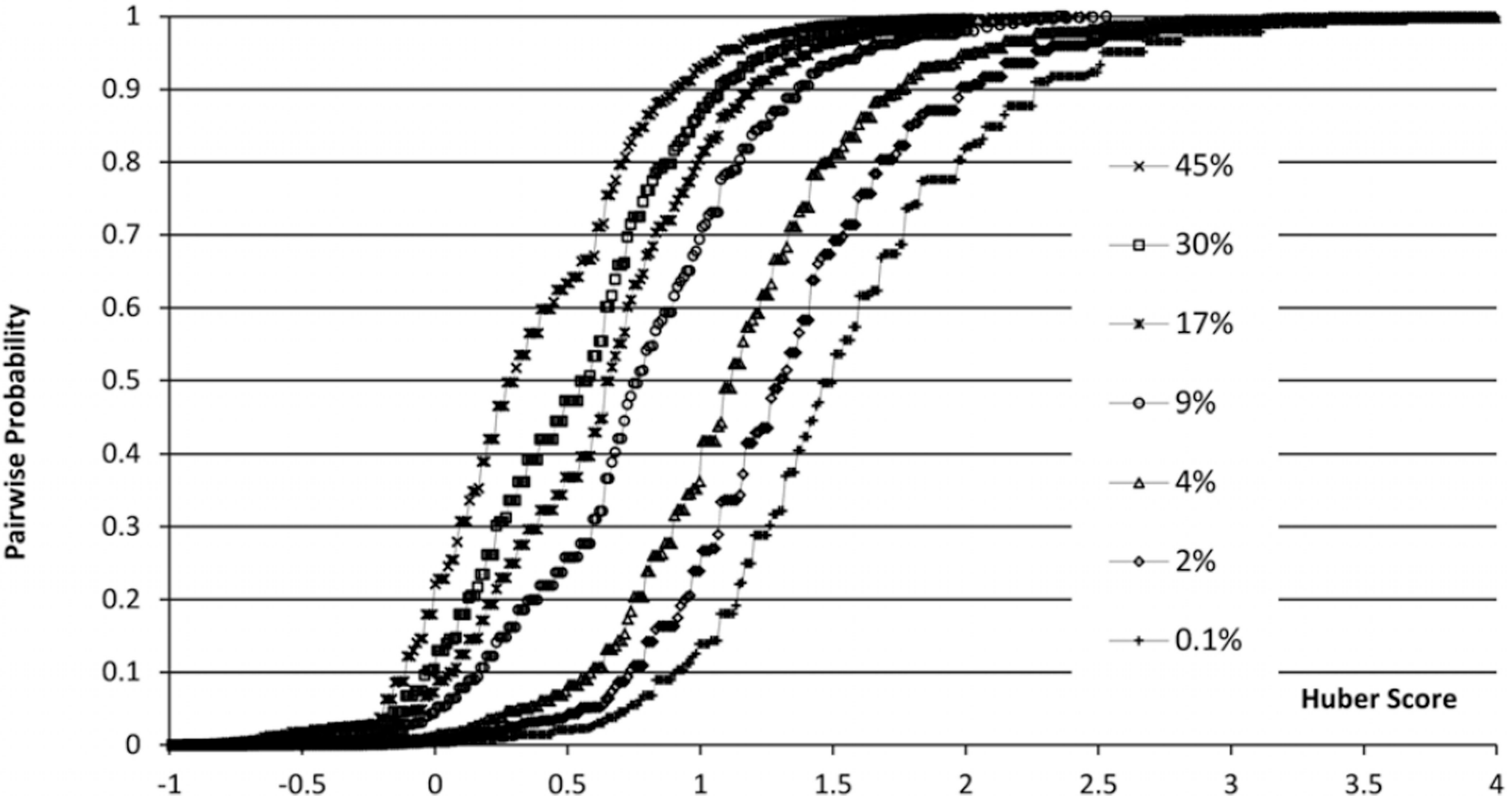
PAV functions of Huber score.

One characteristic of the PAV algorithm is that the function *p(s)* is highly dependent on the relative distribution of positive and negative samples in the input data set. Therefore, only the PAV function obtained from an appropriately constructed input data set is applicable for the namespaces with similar distribution of positive and negative data points. To simulate the namespaces with various distributions of positive and negative pairs, a number of input data sets at different positive portions are constructed by randomly sampling the initial positive and negative data sets. Figure 4 shows the PAV functions derived from input data sets with different positive pair proportions, which enable the Huber similarity scores to be converted to pairwise probabilities in the range of 0~1 for different namespaces. After the positive pair proportion is estimated for a namespace, an appropriate PAV function is selected to convert Huber scores to pairwise probabilities.

**FIG. 5:**
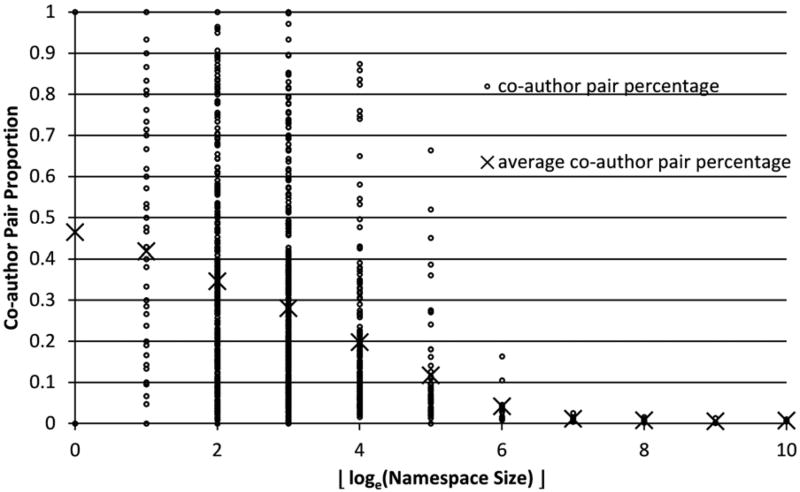
Coauthor pair proportion and name space size (x-axis shows the floor of natural logarithm of namespace size).

Of course, it is hardly feasible to know the percentage of positive pairs for a given namespace before clustering is conducted for it. Instead of pursuing the precise positive proportions, we use the most significant feature we employed in the machine-learning process: the coauthor feature. The pairs of documents which share at least one common coauthor are counted as the approximate number of positive pairs. This method produces reasonable estimates of positive pair proportion for many namespaces. There are some exceptions. For example, some authors publish mostly without any coauthors. The common coauthor method then tends to capture fewer positive pairs than the reality. Another exception would be that two different authors within the same namespace may publish with different coauthors who happen to share the same name, which may mislead the coauthor method into overcounting the positive pairs. To handle these difficult cases, the measurement of positive pair percentage is adjusted by taking the namespace size into consideration. Normally, a larger namespace contains more individual authors, which results in a smaller proportion of positive pairs. To characterize the relationship between positive pair probability and namespace size, the percentage of common coauthor pairs is measured for about 2,500 sample namespaces of different sizes. These samples are grouped by the integer floor of the natural logarithm of their sizes and average positive pair percentage is taken for each group. Their relationship is demonstrated in Figure 5. Although individual coauthor pair percentages are distributed widely within each size group, the average percentage is steadily decreasing with increasing name space size.

In practice, the coauthor pair percentage (*CPP*) measured for a particular namespace is checked against the average coauthor pair percentage (*ave.CPP*) for the corresponding size group. If the measured coauthor pair percentage deviates from the average percentage more than some threshold (Δ is selected as 0.2 empirically), a preset percentage based on the average percentage is used instead as the prior probability in choosing an appropriate PAV function.

$$\text{Positive Pair Probability}\;=\{\begin{array}{cc} \text{ave}.\text{CPP}\times (1-\Delta ), & \text{CPP}<\text{ave}.\text{CPP}\times (1-\Delta )\\ \text{ave}.\text{CPP}\times (1-\Delta ), & \text{CPP}>\text{ave}.\text{CPP}\times (1+\Delta )\\ \text{CPP}, & \text{otherwise} \end{array}$$

### Correcting Transitivity Violations

The methods in our disambiguation system can generate precise pairwise probabilities to faithfully gauge the relationship among most PubMed citations by assigning high probabilities to true positive pairs and low probabilities to true negative pairs. However, due to the imperfect information in some citations, the pairwise probabilities of the pairs involving these citations may falsely deviate from their true values. False-negative pairs, whose calculated probabilities are lower than their true values, and false-positive pairs, whose calculated probabilities are higher than their true values, can cause splitting and lumping errors (Torvik & Smalheiser, 2009), respectively.

False-negative pairs usually involve those citations created decades ago which suffer from missing information in such key fields as affiliation, abstract, etc. As a result, these empty fields produce low similarity scores and hence low pairwise probabilities. Another phenomenon leading to artificially low pairwise probabilities is the segmentation of an individual author’s publication record into different areas, due to a researcher’s education in multiple institutes, change of research interest, or collaboration with different groups of researchers. It is difficult to connect publications from the same author but with different affiliations, coauthors, and research areas.

False-positive pairs usually have two individual authors with similar names who happen to work in the same institute, or share some common research interest, or publish with some common collaborators. The citations from such authors are likely to produce high pairwise probability, while they are different individuals. Previous work (Torvik & Smalheiser, 2009) shows that splitting errors happen more frequently than lumping errors, which suggests that there exist more false-negative pairwise probabilities than false-positive pairwise probabilities. To handle false-negative pairwise probabilities, correction is executed upon triplet transitivity violations:

$$p_{\text{ij}}+p_{\text{jk}}-1>p_{\text{ik}}+\delta ,\text{and }p_{\text{ik}}\leq p_{\text{ij}},p_{\text{jk}}$$

Here δ > 0 is a tunable parameter. The violation occurs in a triangle consisting of three citations (*i, j, k*), in which the high probabilities of the citation pairs (*i, j*) and (*j, k*) suggest that these three citations belong to the same author, while the low probability for the pair (*i, k*) indicates otherwise. To correct this contradiction in a violating triangle, high probabilities are decreased and the low probability is increased while minimizing the overall probability variation (Torvik et al., 2005). This solution is reasonable because it penalizes potential false-positive probabilities and boosts potential false-negative probabilities simultaneously. Due to the observation that false-negative probabilities are more probable than false-positive probabilities, a weighting factor is designed to control the variation by favoring false-negative probabilities. A typical triangle correction lifts the low probability at the cost of lowering the high probabilities. However, the upper bound limit of the renewed value of the low probability *p_ik_* is *p_ij_* + *p_jk_* − 1, which may not be boosted enough in some cases. On the other hand, some true positive probabilities may be overcompromised if they are involved in multiple triangle violations. As a result, some false-negative probabilities are underboosted and some true positive probabilities are overreduced, which fail to capture some potential correction and disambiguation opportunities.

To improve the efficiency of triangle correction, a simplified correction scheme is applied in our work. Instead of reaching minimal overall probability variation, we leave the high probabilities in a violating triangle unchanged while boosting the low probability:

$$p_{\text{ij}}^{\prime }=p_{\text{ij}}$$

$$p_{\text{jk}}^{\prime }=p_{\text{jk}}$$

$$p_{\text{ik}}^{\prime }=p_{\text{ij}}*p_{\text{jk}}$$

Since *p_ij_* * *p_jk_* ≥ *p_ij_* + *p_jk_* − 1, the low probability is assigned with a new value $p_{\text{ik}}^{\prime }$, which represents a more powerful boosting. On the other hand, high probabilities are allowed to keep their original values because the high-precision machine-learning method in capturing same-author relationship helps to minimize the occurrence of false-positive pairs. In previous work (Torvik et al., 2005), some false-positive pairs occur for authors with different first names while sharing a common initial. We automatically regard such pairs as absolute negatives based on the name compatibility check. By strictly checking name compatibility, the name-incompatible true negative pairs are also prohibited from correction and their low probabilities are protected. With name-incompatible pairs excluded from correction, the amount of violation checking is greatly reduced, especially for large namespaces. Thus, this simple method improves both effectiveness and speed of correction.

### Clustering Framework

Based on pairwise distance, agglomerative clustering methods have proven effective in numerous name disambiguation techniques (Torvik & Smalheiser, 2009). This clustering method examines the connectivity of all clusters (*C*) within a namespace and merges documents with short distance (high similarity) to maximize the overall clustering score for the namespace:

$$\text{clustering score }(C)=\prod_{i\in C,j\in C}{p_{\text{ij}}}^{\delta_{\text{ij}}}{\left(1-p_{\text{ij}}\right)}^{1-\delta_{\text{ij}}}$$

Here δ_*ij*_ = 1 if document *i* and *j* belong to the same cluster, and δ_*ij*_ = 0 otherwise. A popular greedy clustering mechanism starts with each document constituting a singleton cluster, which is labeled by the author name instance in that document. Then clusters are allowed to merge if it contributes positively to the matching score for the clusters:

$$\text{MS}(C_{1},C_{2})=\text{matching score }(C_{1},C_{2})\;=\prod_{i\in C_{1},j\in C_{2}}p_{\text{ij}}/(1-p_{\text{ij}})$$

If all the pairwise probabilities *p_ij_* across two clusters *C*_1_ and *C*_2_ can lift the matching score between *C*_1_ and *C*_2_ above threshold 1, these two clusters are allowed to merge into a new cluster. The name label of the new cluster is inherited from the more specific name label of the old clusters. For example, in the namespace of SMITH+J, the new cluster merged from two clusters labeled as *J. Smith* and *John Smith*, respectively will be labeled as *John Smith*, since every document in the new cluster is considered to be authored by *John Smith* after the merging process determines *J. Smith* in one cluster is *John Smith* in the other cluster.

As a bottom-up approach, the quality of the agglomerative clustering method is significantly impacted by the behavior during the initial clustering stages, during which small clusters start to develop and determine the future clustering path. Therefore, it is critical to schedule the merging process among clusters effectively. Our clustering algorithm has two mechanisms to facilitate creating more accurate clusters at earlier stages.

### Name information

Atypical namespace, especially a large namespace consisting of multiple individual authors, contains documents with various name information. Within a namespace, while sharing common family name and first name initial, the name instances could have different or absent first names, different or absent middle names or initials. Based on the assumption that most individual authors normally keep a consistent name representation through their publication records, a clustering priority scheme is designed to coordinate the merging process between two clusters properly, as shown in Table 3.

The higher clustering priority is given to two clusters with better odds of belonging to the same person based on the matching condition of their first and middle name information, and author’s first name is considered of more significance than the middle name in setting priorities due to its higher importance and availability in publications. This clustering priority scheme is also synchronized with the creation of new cluster name labels, which involves less ambiguity in the clustering at higher priority. This scheme helps to create high-quality clusters at earlier clustering stages based on the original name information with little interference from the clustering algorithm. By checking the name information, clusters with incompatible name labels are prohibited from merging.

Previous work has proposed similar methods to handle name variants, such as rules in bootstrapping (Levin et al., 2012), and use of author name similarity profiles (Torvik et al., 2005). Compared to these techniques, our name compatibility scheme works in a more systematic fashion in name disambiguation. In addition to enforcing a name compatibility check on the pairs of citations, we also apply a name compatibility check at the cluster level. When citations with compatible name variants are attributed to a new cluster, the new cluster is assigned with the more specified form of name variant, which is compared with other cluster-level name information for further clustering opportunities. A name compatibility check at the cluster level helps to recover missing name information within clusters and enables efficient cluster merging by avoiding the need to check cross-cluster pairs of documents.

### Probability level

Another important issue in clustering quality is to choose the best clusters for merging. Even at the same clustering priority, there could be multiple candidate clusters to be selected for critical early merging. An effective policy reinforced in our clustering algorithm is to schedule the merging between clusters based on the value of their cross-cluster probabilities and choose the pair of clusters with the best matching score to merge, as shown in the following algorithm:

**function** *Cluster*(*D, prob*)
         **inputs:** *D*, the set of all the citations from a namespace
                 *prob(a, b)*, the pairwise probability for the name-compatible pair
                 of citations *(a, b)* in *D*×*D*
         **output:** *C*, the set of clusters consisting of citations in *D*
*C* = {{*x*}|*x* ∈ *D*}, each initial cluster consisting of one citation *x*
**iteration** {
**for** *p* in [1, 2, 3]
        **for** *CT (clustering threshold)* in [0.9, 0.8, …, 0.1, 0]
                **foreach** cluster *i* in *C*:
                        **foreach** cluster *j* in *C* (*i* ≠ *j* and *priority(i, j)* == *p*):
                                **if** ∃ *prob(a, b)* < *CT, a*∈*i, b*∈*j*: **break**
                                **else:** compute *MS(i, j)*
                        in all *j’s* with *MS(i, j)* > 1, select *J* = *argmax_j_MS(i,j)* and
                        merge cluster pair (*i, J*) in *C*
}
**return** *C*


For each priority level, the clustering threshold is decreasing from 0.9 down to 0 at the step of 0.1. At each threshold level, cluster merging is allowed to occur under the condition that all the pairwise probabilities across the clusters must be above the threshold. At a particular clustering threshold, the pairs with all the intercluster probabilities above the threshold and matching scores above 1 are considered for merging, with the pair with the highest matching score selected to be merged. The threshold starts from 0.9 initially, which permits the clusters with high intercluster connection to be considered for merging. The threshold is decreased gradually in stages, which allows clusters with lower connection to merge. One complete iteration works through priority 1, 2, and 3 in order, and the whole clustering process ends when no more merging opportunities can be found.

Final clustering results are often dependent on how the clustering algorithm starts initially. To add randomness to our clustering framework, the order of the documents can be scrambled to create a number of random initial states, which drive the same clustering algorithm and produce different sets of clustering results. The sets of results are compared with each other by their overall clustering scores, and the highest scoring result is selected as the final clustering.

## Results

### Disambiguation Accuracy

All the PubMed authors are processed with our disambiguation system and updated weekly. Currently PubMed collects more than 22 million citations, which have 76 million author name instances. About 4 million namespaces are created from all the author instances, and about 2.5 million namespaces owning multiple citations are subject to name disambiguation. In total, 10.2 million clusters are produced by the disambiguation process, and the average number of citations per individual author is 7.3, which is similar to other studies. We apply a variety of methods to evaluate the disambiguation accuracy of our clustering results: (1) measure the error rate of random pairwise samples; (2) measure the precision and recall of clustering results against manually curated author profiles; (3) measure the split error with gold standard data (self-reference and common-grant).

Previous work (Torvik & Smalheiser, 2009) measures lumping error (merging citations by different authors to the same cluster) and splitting error (separating citations by the same author into multiple clusters) to evaluate the disambiguation performance. To measure the overall error rate, the pairwise lumping error rate and splitting error rate are defined:

$$\text{PLER }(\text{pairwise lumping error rate})\;=\frac{\#\text{ of incorrectly clustered pairs}}{\#\text{ of all clustered pairs}}$$

$$\text{PSER }(\text{pairwise splitting error rate})\;=\frac{\#\text{ of incorrectly unclustered pairs}}{\#\text{ of all unclustered pairs}}$$

Then the overall error rate is evaluated by combining both lumping and splitting error rates based on their distribution:

$$\text{overall pairwise error rate}=\frac{\#\text{ of all incorrect pairs}}{\#\text{ of all pairs}}\;=\text{PLER}*\text{clustering probability}\;+\text{PSER}*\text{unclustering probability}$$

In addition to the clustering performance metrics, we also define precision and recall as follows:

$$\text{precision}=\frac{\#\text{ of correctly clustered pairs}}{\#\text{ of clustered pairs}}$$

$$\text{recall}=\frac{\#\text{ of correctly clustered pairs}}{\#\text{ of true}-\text{positive pairs}}$$

To evaluate our disambiguation performance and compare to another state-of-the-art method, over 2 million pairs are sampled from Authority 2009 data (Torvik & Smalheiser, 2009), and compared against the same set of pairs from our disambiguation results. All possible pairs within a namespace are considered as candidates for sampling. For a namespace of size *N*, there are in total *N*×*(N−1)/2* possible pairs. A namespace with more than one citation is randomly sampled at a probability in proportion to its total possible pair counting. A sample pair is either clustered (*C*) or unclustered (*U*) by the clustering algorithm. Our results agree with Authority 2009 data on about 83% of the samples, while making different decisions on the remaining 17% of samples, as shown in Table 4.

Random samples are extracted from the four groups of samples for validation by human reviewers. To ensure a fair comparison, these samples were re-disambiguated within the same namespace but only with the citations dated until 2009. Each sample is first examined by two reviewers with all the information that a reviewer can obtain from all possible information sources to independently determine if the pair of citations belong to the same author. If both reviewers make the same decision for a sample, the validation ends with that consensus judgment. For those samples without consensus, the result depends on if one reviewer can persuade the other reviewer to reach agreement. If the reviewers still disagree, the pair will be arbitrated as unclustered.

Since consensus is reached for the samples in group CC (pairs clustered in both Authority 2009 and our result) and group UU (pairs unclustered in both Authority 2009 and our result), the clustering decisions for those samples highly match the human judgments. This is proven by reviewing 50 random samples from group CC and 50 random samples from group UU. Initially, the reviewers disagreed on 6% and 2% of samples for CC and UU groups, respectively. After discussion the disagreement rates dropped to 2% for CC group while UU group remained the same. Compared with the reviewers’ labeling, the observed error rates of our clustering are 2% and 6% in group CC and group UU, respectively, as shown in Table 5.

However, in group CU (pairs clustered in Authority 2009 but unclustered in our result) and group UC (pairs unclustered in Authority 2009 but clustered in our result), there is a higher level of error. For 100 samples in group CU and 100 samples in group UC, initially the reviewers did not agree on 23% and 16% of samples, respectively. Discussions between reviewers reduced disagreement rates to 5% and 4% for CU and UC groups, respectively, which were eventually solved through examination and majority voting by all participating reviewers. Based on reviewers’ labeling, our clustering outperforms Authority 2009 in group CU while underperforming in group UC. As a result, pairwise splitting error rate (PSER) is 9.3% for Authority 2009 and 23.1% for our result, while lower pairwise lumping error rate (PLER) is 12.5% for Authority 2009 and 3.2% for our result, respectively. The performance on the 100 samples for each of CU and UC groups comparing our clustering and Authority 2009 is examined with one-tailed Sign test (Mendenhall, Wackerly, & Scheaffer, 1989), which gives *P* values of 0.097 and 0.242 for CU and UC groups, respectively.

Splitting errors contribute 7.7% and 1.8% to the final error rates for our clustering and Authority 2009, respectively, while lumping errors contribute 2.2% and 10.1%. Since group CU is almost 10 times the size of group UC, our clustering reduces the overall pairwise error rate to 9.9% from 11.9% for Authority 2009. Based on the pairwise error results, we also compute precision and recall scores for both Authority 2009 and our clustering. Compared to Authority 2009, our clustering improves precision while lowering recall. Overall, the F-score increases to 92.9% from 92.2% for Authority 2009.

In addition to evaluation based on random samples, researchers also propose computing precision, recall, and F-scores by comparing reliable reference authorship records with clustering results generated by a disambiguation process (Kang et al., 2009; Ferreira et al., 2010; Levin et al., 2012). With knowledge of all the publications by a particular author (*gold cluster*), a cluster is selected as the *matching cluster*, which among all the computed clusters has the most overlapping publications with the gold cluster, and if a tie occurs, choose the one with the fewest nonoverlapping publications. Then pairwise precision, recall, and F-score are defined:

$$P(\text{gold},\text{matching})=\frac{\left|\text{pairs}(\text{gold})\cap \text{pairs}(\text{matching})\right|}{\left|\text{pairs}(\text{matching})\right|}$$

$$R(\text{gold},\text{matching})=\frac{\left|\text{pairs}(\text{gold})\cap \text{pairs}(\text{matching})\right|}{\left|\text{pairs}(\text{gold})\right|}$$

$$F(\text{gold},\text{matching})\;=\frac{2*P(\text{gold},\text{matching})*R(\text{gold},\text{matching})}{P(\text{gold},\text{matching})+R(\text{gold},\text{matching})}$$

We collected reference authorship information by examining more than 200 researchers in the clinical medicine field from the preliminary lists of Highly Cited Researchers (http://ip-science.thomsonreuters.com/hcr/clinical_medicine.xlsx). Based on availability, some profiles of these researchers are downloaded from various sources, including www.researchgate.net (an online research profile hub based on researchers’ own contribution), Harvard Catalyst Profiles (http://catalyst.harvard.edu/, an academic profile database created with aid of faculty members), COS Pivot (http://pivot.cos.com, a commercial profile database editorially created from publicly available content, university websites and user input), and www.researcherid.com (online profiles managed by researchers with a unique identifier). However, these sources also suffer from inaccurate bibliographies for some authors. Institutional databases (e.g., Harvard Catalyst) may not collect publications written at other institutions, and the COS Pivot database divides some authors’ publications into separate profiles by the different institutions, with which the same author has been affiliated. On the other hand, some authors may be associated with publications that are not authored by them (e.g., more than half of the publications listed under Peter M. Schneider’s ResearcherID do not list his name as author). By comparing and reconciling bibliographic information from different sources, we created gold standard publication records for 40 highly cited researchers complemented with their individual homepage information, and used them as reference clusters for evaluation.

With reference clusters of the 40 researchers, we compare precision, recall, and F-scores in Table 6 for our clustering results against Authority 2009. Our clustering results are generated for the namespaces including the 40 author names based on the citations until 2009. The average precision of our clustering registers a 0.6% increase over Authority 2009 while the average recall is 0.3% lower. The overall F-score is improved by 0.4% over Authority 2009. To test if these differences are statistically significant, we chose the Wilcoxon signed rank test (Neuhäuser, 2012), a nonparametric test examining the magnitudes and signs of the differences between paired observations. The Student’s *t*-test is not applicable because the samples fail the Shapiro–Wilks normality test (Neuhäuser, 2012). With the one-tailed Wilcoxon test, the *P* values for precision, recall, and F-score are 0.015, 0.326, and 0.136, respectively. The test confirms a statistically significant precision improvement in our clustering over Authority, while the loss of recall is not statistically significant at the 0.05 level. As a result of the combined effects, the average gain of F-score is not statistically significant. Additionally, to evaluate the impact of name compatibility check on the disambiguation performance, we conduct disambiguation for the 40 researchers by removing first name information. Consequentially, more lumping errors and fewer splitting errors occur, with average precision and F-scores dropping from 95.7% to 87.9% and from 93.4% to 89.0%, and recall increasing from 92.3% to 92.7%, respectively.

We also collected individual publication records from NIH researchers (many in fellowships) who had at least two publications before 2009. In total, 47 profiles were collected and are employed here as gold standard clusters for evaluation. Compared to Authority 2009 results, the average pairwise precision, recall, and F-scores of our clustering improve by 2.0%, 2.3%, and 2.2%, respectively. Compared to the highly cited researchers, our NIH researchers have significantly smaller numbers of publications (most <100 papers). Small gold standard clusters tend to produce higher variance in performance numbers, and the performance gains are not statistically significant.

In addition to gold standard author profiles, we also evaluated the pairwise error rate with two other gold standard data sets: self-reference pairs and grant participants. By analyzing citation data in PubMed Central (PMC), more than 2.7 million pairs of citations were verified as same author by self-citation. Some pairs of self-citation papers share multiple common authors, and each coauthor is converted to a sample pair for its own namespace. In total 4.7 million such pairs constitute a high-quality gold standard data set for true positive pairs as it is rare for one author to cite another same-name author and our name compatibility check removes incompatible author names automatically. Our results fail to cluster 3% of all these pairs, while 2.1% are unclustered in Authority 2009. The second gold standard test data are created from analyzing over 173 thousand distinct grants in over 1.3 million citations in PubMed. With name compatibility check applied on papers sharing common grant information, about 23 million pairs of common-grant citations are created for all the common authors from those pairs. For a pair of citations sharing multiple grants, only one pair is created. Authority 2009 splits 7.4% of all the pairs, while our result splits 8.5% of all the pairs.

Both self-reference and common grant information are high-quality features indicating the same author relationship; therefore, these data sets demonstrate much lower splitting error rates than random samples. In self-reference data, there is a strong tendency for a pair of papers to share a common topic, as well as coauthors. On the other hand, in large grants, different citations by the same author may be published with completely different collaborators, and share fewer features (e.g., different affiliations), which may lead to lower pairwise similarity and higher splitting error rates.

## Discussion

There are still some cases that our author name disambiguation framework cannot handle correctly, for various reasons, such as unavailability of key information, imperfect data, and noise in the data, among other obstacles. It is necessary in the name disambiguation task, as a practical solution, to consider the tradeoff between different aspects of performance. Compared to other name disambiguation methods, our framework commits to minimizing disambiguation errors while focusing on limiting lumping errors. To improve the user experience with our information retrieval system, precision is very critical for delivering relevant search results. In the case of name disambiguation, lumping errors (although increasing recall) hurt the user experience by mixing citations from different authors in the same cluster and therefore impact search efficiency negatively: (1) the clusters with lumping errors are larger than necessary and may cause a prohibitively long list of retrieval results, (2) citations from different authors may compete for the top ranks, which increases the time for the user to find relevant citations and lowers the chance of success. On the other hand, with a good retrieval interface, some splitting errors may be tolerated by a user, especially when the splitting errors are due to the same author publishing with different coauthors in different areas. A user who queries an author name is usually interested in retrieving closely related citations on similar topics. For such users, a splitting error is a relatively minor issue when the retrieval interface displays the citations from the same cluster in decreasing order of similarity to the query citation. The critical top ranks are still occupied by the most similar citations and are most likely to satisfy the user’s query, which is shown by the improved click through rate of PubMed users on these queries (reported here).

Considering these performance issues, our disambiguation framework takes precautions to minimize lumping errors in the whole process. As shown in Table 4, our clustering only clusters 66.6% of all the sample pairs, while Authority 2009 clusters 80.4%. Most of the gap consists of the pairs clustered in Authority 2009 but separated in our clustering (CU group). In fact, our clustering achieves lower error rate (43%) than Authority 2009 (57%) within this group of pairs. Consequently, our clustering increases overall precision to 96.8%.

One of the important factors leading to lower clustering level and higher precision in our clustering is the strict name compatibility check enforced in the clustering algorithm. Due to lack of a standard for name information among publishers, it is a daunting task to analyze all the author name instances in PubMed correctly. For the same author, there could be various representations in different journals and in different languages. Normally, PubMed employs a last/first/middle name template for author name information. However, due to heterogeneous data sources, an author name could be represented in different ways: first name reduced to initial, and/or, missing middle name information. There are also groups of authors whose naming systems are more complicated than simply last/first/middle names, such as Arabic and Indian names. Regardless of an author’s cultural background, PubMed tries to parse the name instance by a last/first/middle name template, which can cause inconsistency among the name representations of the same author. For some international authors, the publishers may encode author names with non-ASCII characters differently and it takes careful Unicode mapping to connect different encodings of the same name. When Unicode mapping is not applicable for non-roman characters, the names may be romanized in multiple ways (e.g., various phonetic systems exist for romanizing Chinese characters).

Serious efforts are required to handle author names properly, and the tradeoff should be considered when balancing the targets of precision and coverage. Previous work favors coverage more than precision by allowing partial matching or low edit distance when comparing author names. This approach maximizes clustering opportunities regardless of the cultural background of authors. However, for some groups of author names (e.g., Chinese names), partial matching or low edit distance may not be as applicable due to the nature of phonetic transcription. To avoid connecting some apparently different authors, we take a different approach by strictly enforcing name compatibility in our system. This feature helps to achieve a significantly lower PLER in our clustering. To improve recall, we also add some frequent low edit distance name variants to the traditional nickname list. Although it is infeasible to take cultural context of names into consideration for all the authors, we plan to implement more accurate name analysis mechanisms to detect an author’s cultural background for the highly populated author groups. With such mechanisms in place, we will be able to compare names based on the naming customs within a cultural group and compute the similarity properly. For example, partial matching and lowedit distance name pairs may be considered as possibly belonging to the same author among the European population, while regarded as different author identities for those of East Asian background.

Some difficult cases are due to the lack of information. Compared to the high availability of Author, Title, and Journal fields, the other two important PubMed fields, Abstract and Affiliation, are available in only about half of the PubMed citations. External information from publishers, the web, and social networks (Kanani, McCallum, & Pal, 2007; Yang et al., 2008; Pereira, Ribeiro-Neto, Ziviani, Laender, Gonçalves, et al., 2009; Torvik & Smalheiser, 2009; Levin & Heuser, 2010) has been shown to help in such cases. Since our current clustering is completely based on PubMed data, the coverage could be improved with external information. Besides the per-document metadata available in current PubMed citations, modern publishers usually provide more per-author details about recent research papers, some of which are highly indicative of author identity, such as full author names, affiliation information per author, and e-mail contact per author. Previous work takes advantage of some of the information available from publisher websites, and shows a positive impact on coverage. We believe such additional information definitely helps in disambiguation and we plan to extract and incorporate more information from external sources in our future development efforts. Some errors arise from imputing affiliation information to all authors on a citation when such information only applies to the corresponding author. More information specific to individual authors would remedy this problem.

### Online Performance

Since May 2012, the PubMed website has provided access to our disambiguation results by retrieving author clusters for author name queries (http://www.nlm.nih.gov/pubs/techbull/mj12/mj12_pm_author_ranking.html). In the past, clicking on an author name would retrieve all the citations containing that name without disambiguation. Now the author name disambiguation feature provides access to the relevant citation cluster for that clicked author name. The retrieved citations are displayed in the descending order of similarity score against the clicked citation, with more similar citations at higher positions.

By measuring user click-through rate (CTR), an analysis of the PubMed user experience shows that the disambiguation feature has considerably increased the rate that users continue to click on the citations retrieved from an author name query, which suggests that the query results are more relevant to their search interests. Before disambiguation became available, upon clicking on an author name in a citation, PubMed users continued by clicking on one of the retrieved citations 34.9% of the time. This is based on an analysis of 1 week of the PubMed log data for April 2012. A similar analysis was performed 3 months later after PubMed started to provide author name disambiguation functionality. One week of PubMed log data for July 2012 showed that CTR increased to 36.9%. In reality, there is up to 1 week of latency between the appearance of new citations and the appearance of their disambiguation results. During this latency, disambiguation results are not available for ~20% of users’ author name clicks. For the 80% of author name clicks where disambiguation results are available, PubMed users continue to click on a result 39.5% of the time compared to 26.3% for the remaining 20% of author name clicks without a disambiguation result.

## Conclusion

We have presented a complete description of our author name disambiguation system for PubMed citations. The evaluation of the disambiguation results demonstrated that our methods perform well on the entire PubMed collection and help to improve the PubMed users’ online experience.

To provide high-quality author name disambiguation results for a large-scale information retrieval system such as PubMed, machine learning and clustering methods were designed with key features to improve precision. With the ability to detect conflicting name variants, the name compatibility scheme facilitates accurate large-scale training data creation and regulates a clustering algorithm to minimize false clustering. Considering namespace size and proportion of positive pairs, the PAV function enables faithful conversion from similarity scores to pairwise probabilities. By creating the most probable initial clustering, our clustering algorithm chooses clustering paths leading to high-quality final results. The accurate author name disambiguation results benefit PubMed users by providing more relevant information to their name queries.

Our future work will focus on the following aspects. Author name information, the key input to our system, is still imperfect with errors and information loss. Considering the variety of cultural backgrounds of PubMed citation authors, a better human name processing system is necessary to recover the name information lost during the data collection stages. Based on an author’s cultural background, this system will process author names more intelligently and faithfully, providing more accurate information for disambiguation methods. Another possible direction of future work is to take advantage of author relationships to construct author networks which can further capture potential clustering opportunities, as shown to be effective in (Bhattacharya & Getoor, 2007). Author networks can transcend namespaces and provide new features to enhance machine learning and the clustering process. The obstacle to applying this technique to PubMed data is the substantial amount of additional computation. An intelligent method is required to locate the most profitable relations by examining current clustering results. Last but not least, non-PubMed information is shown to have a significant positive impact on disambiguation quality. In addition to correcting errors in PubMed citations, information extracted from publishers can help complement the per-author information which is now lacking in current PubMed data. An author’s personal webpage is another rich source of additional information which may provide the most up-to-date bibliography and help indicate personal identity.

## Figures and Tables

**TABLE 1:** Availability of PubMed citation fields.

Field	Title	Affiliation	Grant	Journal	Abstract	Substance	MeSH	Author	Date
Available	100%	53.2%	8.5%	100%	48.7%	48.5%	91.3%	97.6%	100%

**TABLE 2:** Computed features from PubMed fields.

Field	Field content	Stopword list	Feature
Title	text string	General	similarity_overall_(similarity_1_)
Affiliation	text string	affiliation	similarity_overall_(similarity_2_)
Grant	text string	General	similarity_overall_(similarity_3_)
Journal	text string	General	similarity_overall_(similarity_4_)
Abstract	text string	General	similarity_overall_(similarity_5_)
Substance	text string	General	similarity_overall_(similarity_6_)
MeSH	text string	MeSH	similarity_overall_(similarity_7_)
Author	text string		similarity_name_(similarity_8_)
Date	numerical		year_diff_ (similarity_9_)

*Note*. The affiliation stopword list is the PubMed general stopword list with addition of common affiliation terms.The MeSH stopword list is the PubMed general stopword list with addition of common MeSH terms.

**TABLE 3:** Name information based clustering priority.

	Name label information per cluster			
	
	First name		Middle name	
		
Priority	Cluster 1	Cluster 2	Cluster 1	Cluster 2
1	Same	Same	Same	Same
2	Same	Same	Middle name	None
3	Full first name	First initial	Compatible	

**TABLE 4:** Comparing clustering results.

		Our clustering	
		
		Clustered (C) 66.6%	Unclustered (U) 33.4%
Authority 2009	Clustered (C) 80.4%	CC: 65%	CU: 15.4%
	Unclustered (U) 19.6%	UC: 1.6%	UU: 18%

**TABLE 5:** Pairwise error rate by human review.

Category (# of samples)	CC (50)	UU (50)	CU (100)	UC (100)	PSER	PLER	Error rate (splitting + lumping)
Authority 2009	2%	6%	57%	46%	9.3%	12.5%	11.9% = 1.8% + 10.1%
Our clustering	2%	6%	43%	54%	23.1%	3.2%	9.9% = 7.7% + 2.2%

		Precision			Recall		F-score

Authority 2009		87.5%			97.5%		92.2%
Our clustering		96.8%			89.3%		92.9%

**TABLE 6:** Pairwise precision, recall, and F-scores for highly cited researchers.

Researcher name (last name, first name)	Our clustering			Authority 2009		
	
	Precision	Recall	F-score	Precision	Recall	F-score
Agarwal, Ashok	0.882	0.405	0.556	0.778	0.991	0.871
Alves, Cintia	1.000	0.960	0.980	0.960	0.960	0.960
Ammenwerth, Elske	0.935	1.000	0.966	0.935	1.000	0.966
Amorim, Antonio	0.887	0.988	0.935	0.848	1.000	0.918
Antman, Elliott	0.974	0.916	0.944	0.965	0.970	0.967
Bates, David	1.000	0.778	0.875	1.000	0.822	0.902
Buring, Julie	1.000	1.000	1.000	1.000	0.984	0.992
Camargo, Carlos	0.915	0.993	0.952	0.743	1.000	0.853
Cannon, Christopher	0.977	0.955	0.966	0.964	0.968	0.966
Carrell, Douglas	0.890	1.000	0.942	0.890	1.000	0.942
Durham, Stephen	1.000	0.946	0.972	1.000	0.843	0.915
Eisenberg, David	0.977	0.630	0.766	0.973	0.463	0.628
Epstein, Ronald	1.000	0.807	0.893	1.000	0.653	0.790
Hellstrom, Wayne	0.908	0.981	0.943	0.901	1.000	0.948
Hood, Kerenza	0.821	0.739	0.778	1.000	0.718	0.836
Hu, Frank	0.977	0.703	0.818	0.983	0.679	0.803
Ioannidis, John	1.000	1.000	1.000	1.000	1.000	1.000
Jorgensen, Niels	1.000	1.000	1.000	1.000	0.941	0.969
Kaptchuk, Ted	1.000	1.000	1.000	1.000	1.000	1.000
Kritchevsky, Stephen	1.000	0.967	0.983	1.000	0.903	0.949
Lako, Majlinda	1.000	1.000	1.000	1.000	1.000	1.000
Leffers, Henrik	0.964	1.000	0.982	0.964	1.000	0.982
Libby, Peter	0.921	0.885	0.903	0.911	0.966	0.938
Manson, JoAnn	1.000	0.962	0.981	1.000	0.993	0.996
Ridker, Paul	1.000	0.977	0.988	1.000	0.995	0.998
Rifai, Nader	1.000	0.966	0.983	0.989	0.994	0.992
Rimm, Eric	1.000	1.000	1.000	1.000	1.000	1.000
Rodriguez Martinez, Heriberto	0.780	1.000	0.877	0.768	1.000	0.869
Roewer, Lutz	0.925	1.000	0.961	0.925	1.000	0.961
Schneider, Peter	0.984	0.923	0.953	0.983	0.792	0.877
Simonsick, Eleanor	0.985	1.000	0.992	0.985	1.000	0.992
Stampfer, Meir	0.992	0.914	0.952	0.990	0.938	0.963
Sunde, Kjetil	0.904	1.000	0.949	0.861	1.000	0.925
Szibor, Reinhard	0.924	1.000	0.961	0.901	1.000	0.948
Ter Kuile, Feiko	0.823	1.000	0.903	0.823	1.000	0.903
Thomson, James	0.946	0.895	0.920	1.000	0.797	0.887
Vincent, Jean Louis	1.000	0.876	0.934	1.000	0.957	0.978
Weiss, Scott	0.971	0.809	0.883	0.973	0.700	0.814
Willett, Walter	0.998	0.938	0.967	0.997	0.991	0.994
Yen, Kathrin	1.000	1.000	1.000	1.000	1.000	1.000
Average ± standard deviation	0.957 ± .057	0.923 ± .123	0.934 ± .083	0.950 ± .071	0.925 ± .125	0.930 ± .076
